# Dose-dependent effects of curcumin on bacterial growth and sperm quality during refrigerated storage of equine epididymal sperm

**DOI:** 10.3389/fvets.2026.1739360

**Published:** 2026-02-09

**Authors:** Lydia Gil, Noelia González, Lydia Horndler, Victoria Luño

**Affiliations:** 1 Departament of Animal Pathology, Universidad de Zaragoza, Zaragoza, Spain; 2 Instituto Universitario de Investigación Mixto Agroalimentario de Aragón (IA2), Universidad de Zaragoza, Zaragoza, Spain; 3 Universidad San Jorge (USJ), Zaragoza, Spain

**Keywords:** acrosomal integrity, antibiotic alternatives, antioxidant, bacterial growth, curcumin, equine sperm preservation, refrigeration, sperm motility

## Abstract

Cooling equine sperm for storage can reduce its quality and functional characteristics, presenting challenges for preservation methods. During refrigeration, spermatozoa are exposed simultaneously to oxidative stress and bacterial contamination, both of which compromise sperm viability and fertility potential. To limit bacterial proliferation, commercial extenders are routinely supplemented with antibiotics, but growing concern about antimicrobial resistance has prompted the search for natural alternatives. This study aimed to explore the effects of curcumin, a polyphenolic compound from *Curcuma longa* with well-documented antioxidant and antimicrobial properties, on refrigerated equine epididymal sperm over 96 h, focusing on its antimicrobial activity and impact on basic sperm quality parameters in an antibiotic-free extender. Sperm samples were collected from 12 stallions and diluted in an antibiotic-free extender containing different concentrations of curcumin: 0 mM (control), 0.125 mM, 0.25 mM, and 0.5 mM. Parameters such as motility, viability, acrosomal integrity, and bacterial growth were evaluated after 1 and 96 h of storage at 4 °C to 6 °C. The results showed no significant effects of curcumin on sperm quality at 1 h. However, after 96 h, higher curcumin concentrations (0.25 and 0.5 mM) reduced motility and viability compared to the control group. Despite this, all tested concentrations significantly inhibited cultivable aerobic bacterial growth after 96 h, with 0.125 mM curcumin offering the most favorable balance between antimicrobial effect and preservation of basic sperm quality parameters. These findings provide preliminary evidence that low concentrations of curcumin may act as a potential complementary or partial alternative to antibiotic use in equine semen extenders, although further studies in ejaculated semen and with more comprehensive functional and microbiological assessments are required before its routine application can be recommended.

## Graphical abstract

**Figure:**
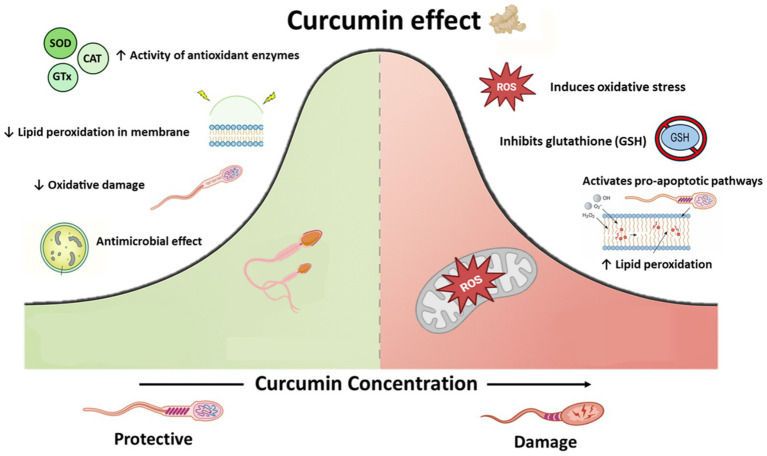

## 1 Introduction

Short-term preservation of equine semen by refrigeration is widely used in assisted reproductive programs due to its relatively low cost, logistical convenience, and acceptable fertility rates following artificial insemination (1). Storage at 4 °C to 6 °C reduces sperm metabolic activity and slows cellular deterioration, thereby extending sperm lifespan during transport and temporary storage (2). However, even under optimized cooling conditions, sperm quality progressively declines over time, limiting the duration during which fertilizing capacity can be maintained (3, 4).

To preserve sperm functional competence during refrigeration, semen is diluted in specialized extenders designed to maintain motility, membrane integrity, and metabolic viability (4, 5). The longevity of cooled semen is influenced by multiple factors, including sperm concentration, initial semen quality, extender composition, dilution and cooling rates, and the presence of antimicrobial agents (6). Despite these measures, refrigerated storage remains a biologically stressful condition for sperm cells.

During cooling, equine spermatozoa are exposed to oxidative stress associated with the generation of reactive oxygen species (ROS), with the superoxide anion being the predominant species produced at low temperatures, which may contribute to membrane damage and functional deterioration during refrigerated storage (7–9). Excessive ROS production can compromise membrane lipids, proteins, and DNA, leading to impaired motility, reduced viability, and altered acrosomal function (7, 10). For this reason, antioxidant supplementation has been explored as a strategy to mitigate oxidative damage and preserve sperm function during storage. A wide range of antioxidant compounds, including enzymatic systems, vitamins, carotenoids, and plant-derived polyphenols, have been incorporated into semen extenders, with variable outcomes depending on species, concentration, extender composition, and storage conditions (11).

In parallel with oxidative stress, bacterial contamination represents a major challenge in semen preservation. Microorganisms may be introduced during semen collection or processing, and even low initial contamination can lead to substantial bacterial proliferation during refrigeration (12). Bacterial growth is associated with the release of lipopolysaccharides and other metabolites that impair sperm motility, membrane integrity, mitochondrial activity, and overall lifespan, ultimately compromising fertility potential (12, 13). In addition, contaminated semen poses a risk of transmitting pathogenic agents to inseminated mares. For these reasons, commercial equine semen extenders routinely contain antibiotics to suppress microbial proliferation and prevent sperm quality deterioration during storage (14).

Despite their widespread use, the antimicrobial activity of antibiotics included in semen extenders is not always sufficient to completely prevent bacterial growth during storage, particularly under prolonged refrigeration. Moreover, their efficacy may vary depending on the initial bacterial load, microbial species, extender composition, and storage conditions (15, 16). In addition, prolonged exposure to certain antibiotics or preservatives has been reported to negatively affect sperm membrane integrity, mitochondrial function, or overall sperm longevity (17, 18). These limitations highlight the need to explore alternative or complementary strategies to control bacterial proliferation in preserved semen (14, 19).

However, the widespread use of antibiotics in animal reproduction contributes to the emergence of antimicrobial resistance, raising concerns in both veterinary and public health contexts. Recent European regulations promote a reduction in prophylactic antibiotic use in animal production, encouraging the development of alternative antimicrobial strategies for semen preservation (20). Consequently, natural bioactive compounds with antimicrobial activity are increasingly being explored as potential substitutes or adjuncts to conventional antibiotics in semen extenders (19).

*Curcuma longa* is a medicinal plant whose principal bioactive constituent, curcumin, is a polyphenolic compound with well-documented biological activity (21–24). Curcumin has been reported to exhibit antioxidant, anti-inflammatory, and antimicrobial properties, including the ability to inhibit bacterial growth through multiple mechanisms (25–29). In reproductive biology, curcumin has been evaluated in several species, where low concentrations have been associated with neutral or protective effects on sperm quality, whereas higher concentrations may exert cytotoxic or pro-oxidant effects (23, 30–33). In addition, both *Curcuma longa* extracts and purified curcumin have demonstrated antibacterial activity against a wide range of microorganisms (34).

In equine reproduction, most studies evaluating microbial contamination and antibiotic alternatives have focused on ejaculated semen, which typically presents higher bacterial loads due to exposure to urethral and preputial microbiota (35). In contrast, sperm recovered from the cauda epididymidis is not sterile but generally exhibits lower initial bacterial contamination compared with ejaculated semen, as previously reported in equine studies (36–38). Nevertheless, epididymal sperm is clinically relevant, as it represents the only source of genetic material in cases of sudden death or inability to ejaculate, and it provides a standardized, antibiotic-naïve model for evaluating the intrinsic effects of novel extender additives without interference from seminal plasma or prior antimicrobial exposure (39–42).

The aim of this study was to evaluate the effects of curcumin supplementation in an antibiotic-free extender on refrigerated equine epididymal sperm, focusing on two complementary aspects: (i) its ability to limit bacterial growth during storage, and (ii) its impact on basic sperm quality parameters, including motility, viability, and acrosomal integrity, over 96 h of refrigeration. By exploring the balance between antimicrobial efficacy and sperm safety, this work seeks to provide initial evidence regarding the suitability of curcumin as a potential complementary or partial alternative to antibiotics in equine semen extenders.

## 2 Materials and methods

### 2.1 Reagents and media

The medium used for semen washing and centrifugation was Equiplus®, an antibiotic-free equine semen extender (Minitüb GmbH, Germany). Curcumin (Sigma-Aldrich, Cat. No. C7727) was prepared as a stock solution by dissolving 1 mg in 1 mL of DMSO. A 1:10 dilution was made to obtain a working concentration of 100 μg/mL, and further 1:2 dilutions were made in PBS to reach the desired concentrations, ensuring that the final DMSO concentration in the sample did not exceed 1%.

For microbiological analyses, two different media were employed depending on the purpose: MacConkey Agar, a selective and differential medium for Gram-negative bacteria, prepared in-house in the university laboratory, and Petrifilm® Aerobic Count plates (3 M, Barcelona, Spain), containing standard methods agar nutrients, a cold water-soluble gelling agent, and a red indicator dye for total aerobic counts.

### 2.2 Collection, processing, and storage of semen samples

Epididymal spermatozoa were obtained from the epididymides of 12 stallions collected at the local slaughterhouse (Mercazaragoza, Zaragoza) and transported to the laboratory at room temperature. Spermatozoa were collected using the retrograde epididymal tail washing technique (19). Briefly, the epididymis was dissected, and a 25 G needle was inserted into the vas deferens and connected to a syringe containing 10 mL of Equiplus® medium without antibiotics at room temperature. Manual pressure was applied to collect spermatozoa into a beaker.

Following collection, initial sperm motility was assessed using the ISAS® computer analysis system (Proiser, Spain). Only samples with motility between 30% and 40% were used. This range was selected to standardize baseline sperm quality among samples and to mimic suboptimal but clinically relevant conditions frequently encountered in epididymal recoveries, thereby facilitating the detection of both protective and detrimental effects of curcumin during storage. Sperm samples were then diluted with Equiplus® to a final concentration of 50 × 10^6^ sperm/ml, centrifuged at 1000 × *g* for 5 min at room temperature, and the supernatant was removed. Each pellet was subsequently resuspended in the four experimental extenders corresponding to the different curcumin concentrations: Control (Equiplus® + 0.5% DMSO), C1 (Equiplus® + 0.5% DMSO + 0.125 mM), C2 (Equiplus® + 0.5% DMSO + 0.25 mM), and C3 (Equiplus® + 0.5% DMSO + 0.5 mM). Finally, the samples were prepared in doses containing a total of 25 × 10^6^ sperm/ml and stored in Eppendorf tubes at 4 °C for up to 96 h.

### 2.3 Sperm quality assessment

Before motility assessment, samples were warmed in a thermoblock at 37 °C for 5 min to allow recovery after cold storage. Sperm motility and movement patterns were objectively assessed using a computerized analysis system (ISAS®; Proiser, Valencia, Spain). For each evaluation, a volume of 4 μL was placed on a microscope slide at 37 °C, with three fields per sample, each containing a minimum of 200 spermatozoa. The set parameters were 25 frames/s, where spermatozoa had to be present in at least 15 frames to be counted.

The parameters evaluated were total motile spermatozoa (TM%), curvilinear velocity (VCL, μm/s), straight-line velocity (VSL, μm/s), average path velocity (VAP, μm/s), curvilinear trajectory linearity (LIN; ratio of VSL/VCL, %) and lateral head amplitude (ALH, μm). Spermatozoa with a mean velocity (VAP) ≥ 20 μm/s were considered motile, whereas those with a VAP < 10 μm/s were considered immotile. Patterns of motile quality were also analysed (40).

**Figure 1:**
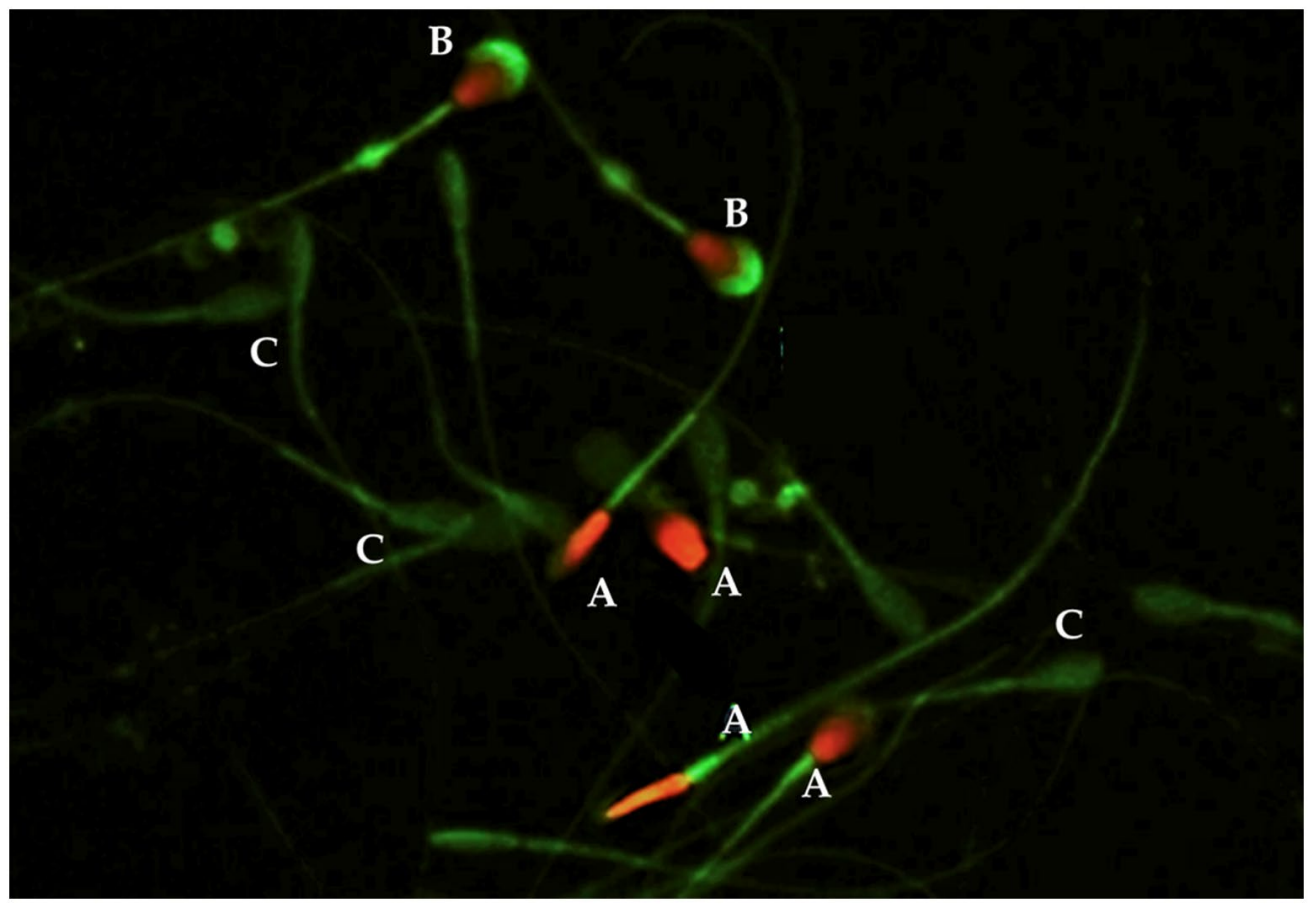
Plasma membrane integrity and acrosomal integrity in equine spermatozoa. Representative images illustrating sperm subpopulations classified according to viability and acrosomal status: (A) Non-viable spermatozoa, (B) Non-viable spermatozoa with acrosomal damage, and (C) Viable spermatozoa with intact acrosome.

Plasma membrane integrity (viability) and acrosomal integrity were jointly assessed by double staining with fluorescein isothiocyanate–conjugated peanut agglutinin (PNA-FITC) and propidium iodide (PI) (43). Samples were incubated with FITC-PNA (1 mg/mL) and PI (500 μg/mL) for 5 min at 37 °C and subsequently fixed in 4% paraformaldehyde. A minimum of 200 spermatozoa per sample were evaluated under a fluorescence microscope (Leica DM2500 LED, Spain). Spermatozoa excluding PI were considered viable, while FITC-PNA labeling of the apical head region indicated acrosomal damage (Figure 1).

### 2.4 Microbiological analysis

To evaluate bacterial growth using two complementary approaches, semen samples were analysed by culture on MacConkey agar plates for the selective detection of Gram-negative bacteria, and on Petrifilm® Aerobic Count plates to estimate overall aerobic cultivable bacterial growth under the incubation conditions used.

For each sample, a 1:10 dilution was prepared by mixing 100 μL of semen with 900 μL of sterile saline solution. From this dilution, 1 mL was inoculated onto MacConkey agar plates using the pour plate technique, specifically aimed at detecting Gram-negative aerobic and facultative anaerobic bacteria. In parallel, 1 mL of the same dilution was inoculated onto Petrifilm® Aerobic Count plates (3 M, St. Paul, MN, USA) following the manufacturer’s instructions to assess total aerobic cultivable bacteria. All samples were processed in duplicate for each method.

Plates were incubated aerobically at 37 °C for 24 h. After incubation, colony-forming units (CFU) were counted, and results were expressed as CFU/mL after correction for the dilution factor. Results obtained from both culture systems were used to compare bacterial growth among treatments and storage times.

Strict anaerobic bacteria were not evaluated, as neither MacConkey agar nor Petrifilm® Aerobic Count plates, under aerobic incubation, are suitable for their detection.

### 2.5 Experimental design

**Figure 2:**
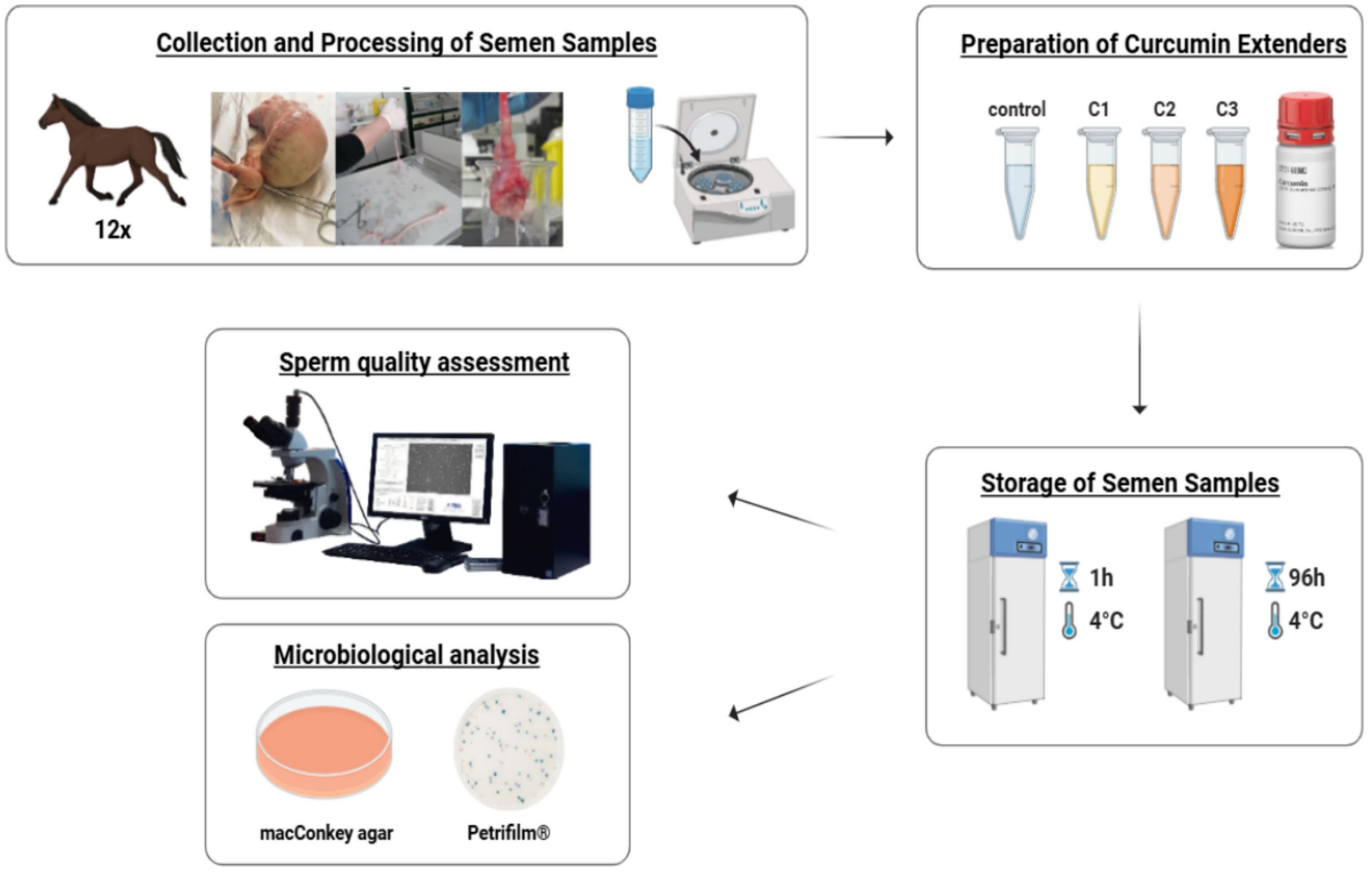
Experimental design. Equine semen was processed with curcumin-supplemented extenders (Control, C1–C3), stored at 4 °C, and analyzed at 1 and 96 h for sperm quality (microscopy and CASA) and bacterial growth (MacConkey agar and Petrifilm® plates).

Sperm quality, including total motility, kinetic parameters, viability, and acrosome integrity, as well as bacterial growth assessed by traditional culture methods and Petrifilm®, were evaluated in relation to the addition of different concentrations of curcumin to stallion sperm extenders. A total of 12 samples (*n* = 12) were analyzed after 1 and 96 h of refrigerated storage at 5 °C. The overall experimental design is presented in Figure 2.

### 2.6 Statistical study

Statistical analysis was performed using IBM SPSS Statistics version 22.0. For each storage time (1 and 96 h), quantitative variables were analysed using a univariate general linear model for repeated measures, with curcumin treatment (Control, C1, C2, C3) as a within-subject factor and stallion as the subject (random) factor. Prior to analysis, model residuals were checked for normality and homoscedasticity. Data are presented as mean ± standard error. When a significant main effect of treatment was detected, pairwise comparisons between groups were carried out with Bonferroni adjustment for multiple testing. Different superscript letters within a row indicate statistically significant differences between treatments (*p* < 0.05).

## 3 Results

### 3.1 Effect of curcumin on sperm quality

**Figure 3:**
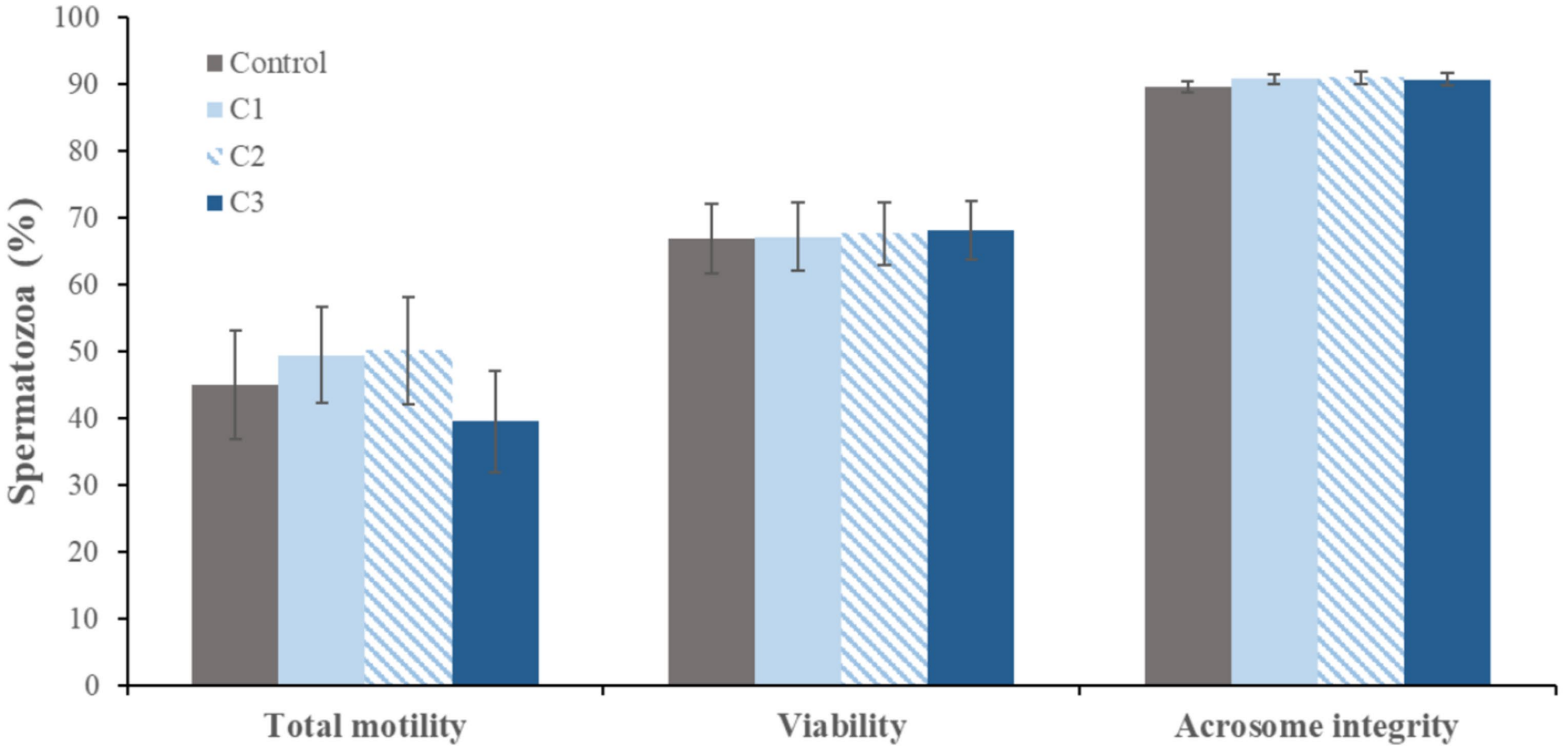
Effect of curcumin on sperm quality parameters at 1 h of refrigeration (mean ± standard error, *n* = 12). Curcumin concentrations: C1 (0.125 mM), C2 (0.25 mM), and C3 (0.5 mM).

**Figure 4:**
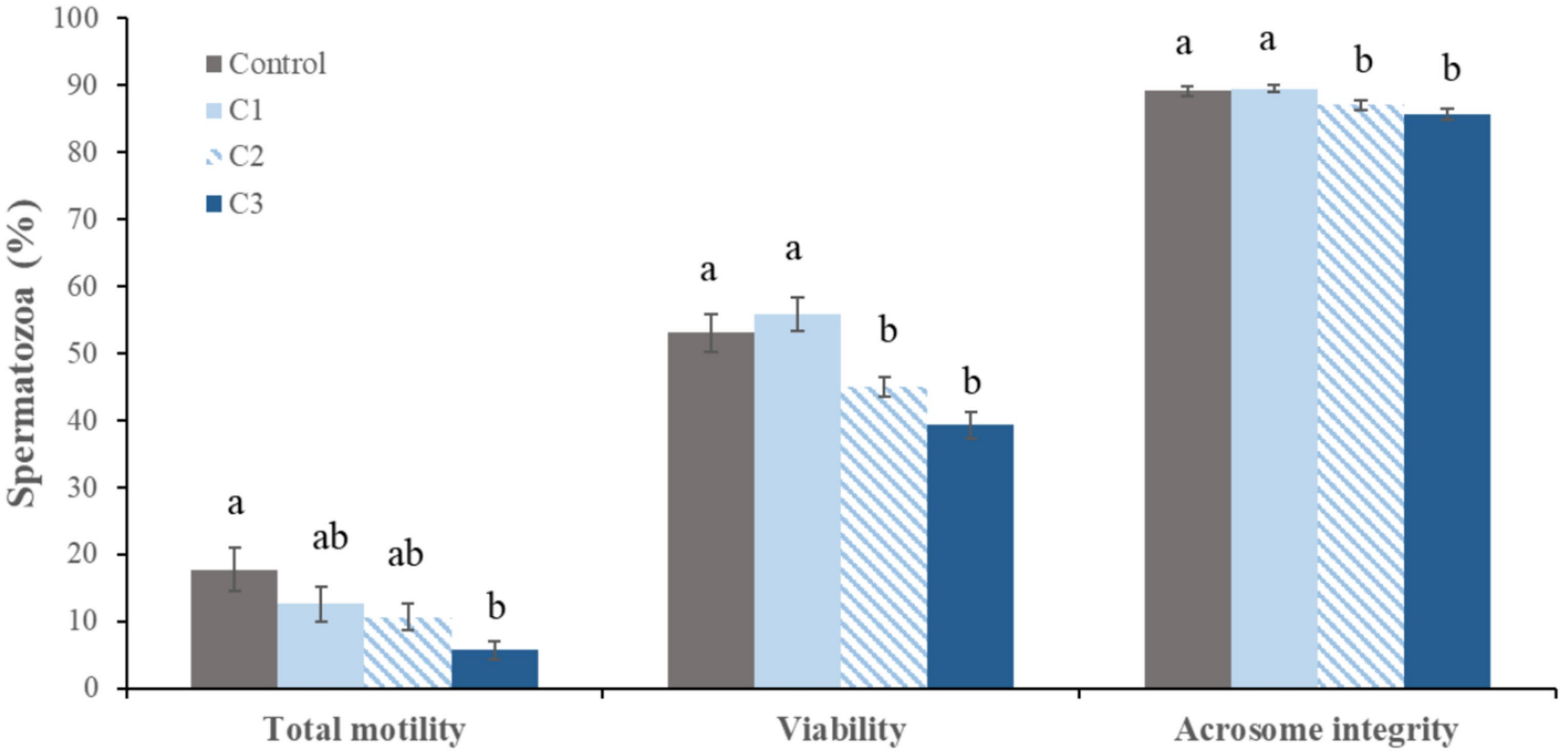
Effect of curcumin on sperm quality parameters after 96 h of refrigeration (mean ± standard error, *n* = 12). Curcumin concentrations: C1 (0.125 mM), C2 (0.25 mM), and C3 (0.5 mM). Significance is denoted by different letters (*p* < 0.05) between treatment groups.

Figures 3 and 4 present the mean values of sperm quality parameters evaluated after 1 and 96 h of refrigerated storage. Figure 3 shows the sperm quality parameters after 1 h of refrigeration, whereas Figure 4 shows the corresponding values after 96 h of storage. After 1 h of refrigeration, supplementation of the extender with curcumin did not result in significant differences in total sperm motility, plasma membrane integrity (viability), or acrosomal integrity when compared with the control group. Although a slight reduction in total motility was observed in samples supplemented with the highest curcumin concentration (0.5 mM; C3), this decrease was not statistically significant.

After 96 h of refrigerated storage, differences among treatments were observed (Figure 4). Samples supplemented with 0.25 mM (C2) and 0.5 mM (C3) curcumin showed significantly lower percentages of spermatozoa with intact plasma membrane and acrosomal integrity compared with the control and 0.125 mM (C1) groups. Total sperm motility was significantly reduced in the C3 group compared with the control, whereas no significant differences were detected between the control and C1 samples.

The extender supplemented with 0.125 mM curcumin (C1) did not significantly affect sperm quality parameters at either evaluation time compared with the control extender.

Analysis of the individual factors included in the statistical model revealed that, at the 1-h evaluation, significant differences were associated exclusively with the stallion factor (*p* < 0.001). In contrast, after 96 h of refrigeration, significant effects were detected for stallion, curcumin concentration, and the stallion × curcumin interaction (*p* < 0.001), indicating that both individual variability and treatment influenced sperm quality parameters during prolonged storage.

### 3.2 Effect of curcumin on sperm motility parameters

The effect of curcumin on motility parameters after cold storage at 4 °C for 1 and 96 h is summarized in Table 1. After 1 h of refrigeration, significant differences among treatments were observed for several kinematic parameters. Curvilinear velocity (VCL) showed higher values in the control and C1 samples than in the C2 and C3 groups. Average path velocity (VAP) showed a similar pattern, with significantly higher values in the control and C1 samples compared with C2 and C3. In contrast, straightness (STR) values were significantly higher in the C2 and C3 groups compared with the control and C1 samples. The C3 group showed higher values for beat cross frequency (BCF) compared with the other concentrations.

**Table 1:** Effect of curcumin on movement quality parameters at 1 h and at 96 h (mean ± standard error, *n* = 12).

T		VCL (μm/sg)	VSL (μm/sg)	VAP (μm/sg)	LIN (%)
1 h	Control	77.30 ± 5.61^a^	36.63 ± 2.71	56.21 ± 4.07^a^	47.91 ± 2.85
	C1	65.12 ± 6.61^a,b^	32.86 ± 3.48	47.20 ± 4.87^a,b^	52.10 ± 3.12
	C2	56.01 ± 5.69^b^	30.28 ± 3.20	40.45 ± 4.59^b^	55.11 ± 3.23
	C3	56.98 ± 6.10^b^	30.10 ± 4.25	38.60 ± 5.35^b^	53.30 ± 3.13
**T**		**STR (%)**	**WOB (%)**	**ALH (μm)**	**BCF (Hz)**
1 h	Control	65.67 ± 2.04^b^	72.87 ± 2.64	3.61 ± 0.21	8.60 ± 0.23^b^
	C1	65.11 ± 4.76^b^	73.58 ± 2.67	3.41 ± 0.20	8.40 ± 0.21^b^
	C2	75.81 ± 2.27^a^	72.47 ± 3.05	3.31 ± 0.26	9.71 ± 0.69^a,b^
	C3	77.82 ± 1.23^a^	66.82 ± 3.41	3.23 ± 0.16	10.67 ± 0.45^a^
**T**		**VCL (μm/sg)**	**VSL (μm/sg)**	**VAP (μm/sg)**	**LIN (%)**
96 h	Control	71.09 ± 9.07^a^	24.30 ± 3.07^a^	37.53 ± 4.12^a^	35.26 ± 3.20
	C1	60.54 ± 5.83^a^	22.27 ± 2.60^a^	33.34 ± 3.07^a^	36.71 ± 2.15
	C2	48.51 ± 6.47^a^	19.06 ± 3.74^a^	28.25 ± 4.37^a^	39.35 ± 4.15
	C3	24.85 ± 2.55^b^	9.65 ± 0.88^b^	15.72 ± 1.28^b^	41.83 ± 4.81
**T**		**STR (%)**	**WOB (%)**	**ALH (μm)**	**BCF (Hz)**
96 h	Control	65.95 ± 3.58	54.72 ± 1.69^b^	3.76 ± 0.40^a^	10.51 ± 1.21^a^
	C1	65.47 ± 4.57	58.72 ± 1.57^a,b^	3.72 ± 0.27^a^	10.13 ± 1.59^a^
	C2	62.28 ± 4.33	58.85 ± 3.50^a,b^	2.04 ± 0.41^b^	5.47 ± 1.16^b^
	C3	64.44 ± 1.96	65.71 ± 3.69^a^	0.85 ± 0.46^b^	1.91 ± 1.17^b^

Different letters (a, b) in the same line and time indicate significant differences (*p* < 0.05). C1 (0.125 mM curcumin), C2 (0.25 mM curcumin) and C3 (0.5 mM curcumin). Different letters (a, b) within the same row and time point indicate significant differences between treatments (*p* < 0.05). VCL: curvilinear velocity; VSL: straight line velocity; VAP: average path velocity; LIN: linearity. STR: straightness; WOB: wobble; ALH: lateral head displacement; BCF: Beat cross frequency.

In relation to other parameters, VSL (straight-line velocity), LIN (linearity), WOB (wobble), and ALH (lateral head displacement), no significant differences were observed among treatments at 1 h of refrigeration.

After 96 h of refrigerated storage, curcumin concentration influenced several motility parameters. Samples supplemented with 0.5 mM curcumin (C3) showed significantly lower values for VCL, VSL, and VAP compared with the control, C1, and C2 groups. No significant differences in velocity parameters were detected among the control, C1, and C2 samples. Control and C1 semen samples showed significantly higher values for ALH and BCF than C2 and C3. Conversely, wobble (WOB) values were significantly higher in the C3 group compared with the control.

Analysis of the statistical model indicated that, at 1 h of refrigeration, the stallion factor had a significant effect on most motility parameters, reflecting inter-individual variability. After 96 h of storage, curcumin concentration and the stallion × curcumin interaction significantly influenced most velocity-related parameters (Table 2).

**Table 2:** Statistical significance (*p*-value) of sperm motility parameters as influenced by study factors (horse effect, curcumin addition, and the horse-curcumin interaction) at 1 and 96 hours of refrigeration.

T	Factor	VCL (μm/sg)	VSL (μm/sg)	VAP (μm/sg)	LIN (%)
1 h	Horse	<0.001	<0.001	<0.001	0.013
	Curcumin	0.011	0.184	0.03	0.287
	Horse * curcumin	0.039	0.513	0.05	0.439
**T**	**Factor**	**STR (%)**	**WOB (%)**	**ALH (μm)**	**BCF (Hz)**
1 h	Horse	<0.001	<0.001	<0.001	0.134
	Curcumin	0.057	0.174	0.433	<0.001
	Horse * curcumin	0.003	0.360	0.629	0.002
**T**	**Factor**	**VCL (μm/sg)**	**VSL (μm/sg)**	**VAP (μm/sg)**	**LIN (%)**
96 h	Horse	0.12	0.004	0.049	0.126
	Curcumin	<0.001	<0.001	<0.001	0.564
	Horse * curcumin	<0.001	<0.001	<0.001	0.643
**T**	**Factor**	**STR (%)**	**WOB (%)**	**ALH (μm)**	**BCF (Hz)**
96 h	Horse	<0.001	0.827	0.607	0.053
	Curcumin	0.863	0.041	<0.001	<0.001
	Horse * curcumin	0.919	0.047	<0.001	<0.001

### 3.3 Curcumin’s effect on bacterial growth

Bacterial growth in semen samples during refrigerated storage was evaluated after 1 and 96 h using two methods: traditional culture on MacConkey agar and Petrifilm® Aerobic Count plates. Both methods were used to estimate cultivable aerobic bacterial growth under the different curcumin concentrations.

**Figure 5:**
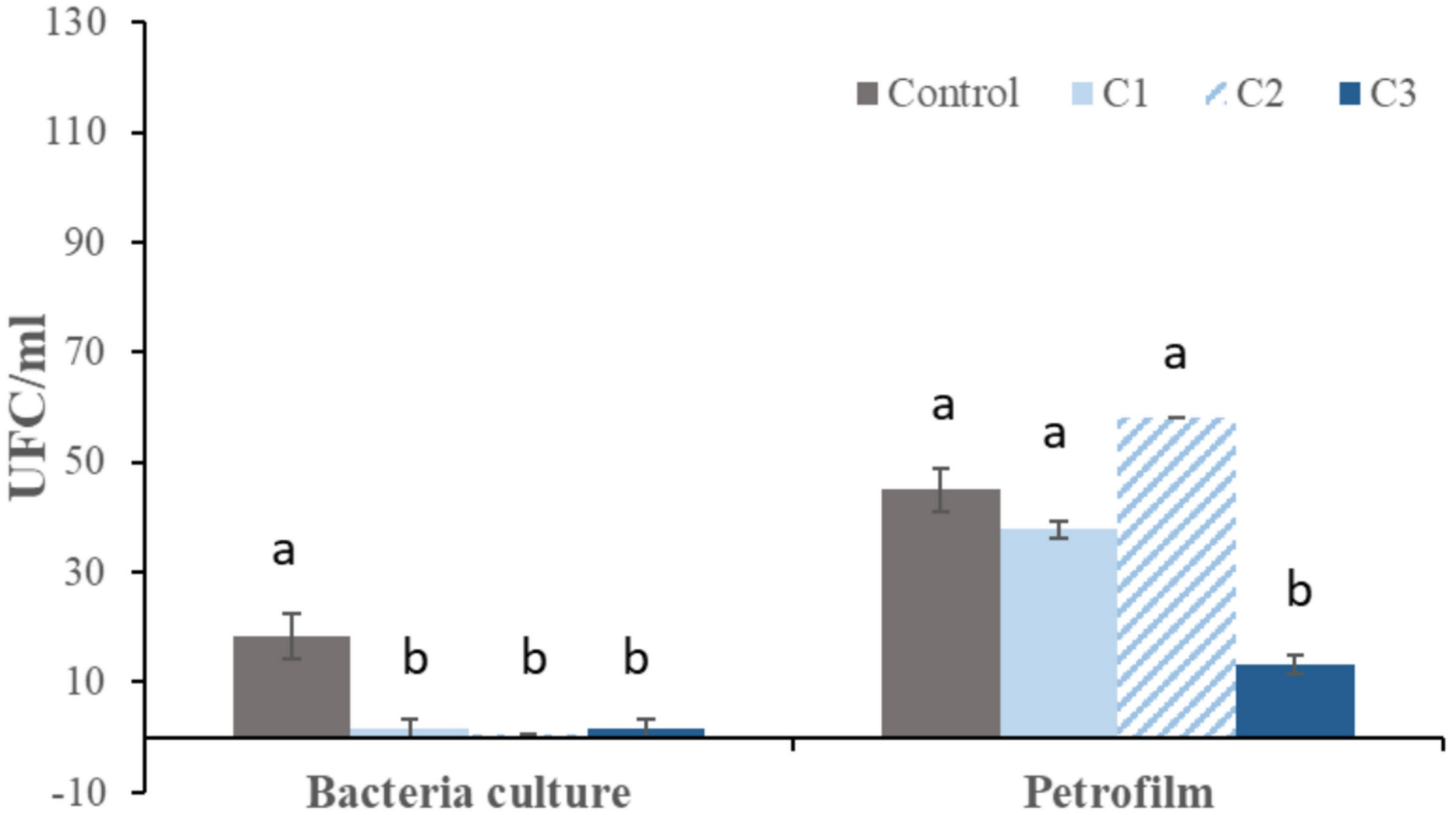
Bacterial growth after 1 h of refrigeration. Bacterial counts obtained by culture on MacConkey agar and by Petrifilm® Aerobic Count plates (Petrifilm). Data are expressed as mean ± standard error (*n* = 12). Curcumin concentrations: Control (0 mM), C1 (0.125 mM), C2 (0.25 mM), and C3 (0.5 mM). Different letters (a, b) within the same method indicate significant differences between treatments (*p* < 0.05).

After 1 h of refrigeration, traditional bacterial culture showed significantly higher bacterial growth in the control samples compared with all curcumin-supplemented groups. No significant differences were observed among the different curcumin concentrations. In contrast, Petrifilm® analysis revealed similar bacterial counts in the control, C1, and C2 groups, whereas samples supplemented with 0.5 mM curcumin (C3) showed significantly lower bacterial growth compared with the other treatments (Figure 5).

**Figure 6:**
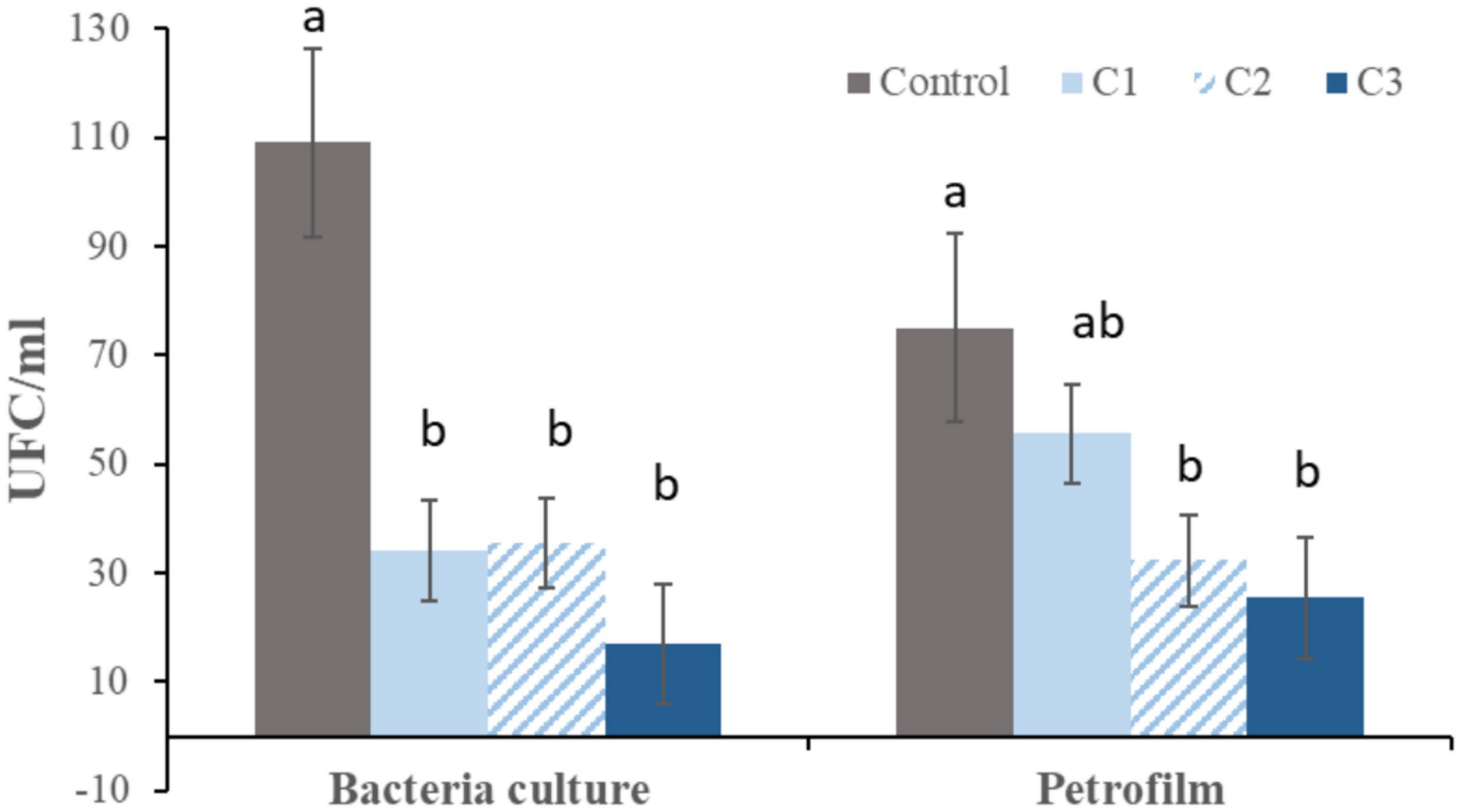
Bacterial growth after 96 h of refrigeration. Bacterial counts obtained by culture on MacConkey agar (bacteria culture) and by Petrifilm® Aerobic Count plates (Petrifilm). Data are expressed as mean ± standard error (*n* = 12). Curcumin concentrations: control (0 mM), C1 (0.125 mM), C2 (0.25 mM), and C3 (0.5 mM). Different letters (a, b) within the same method indicate significant differences between treatments (*p* < 0.05).

After 96 h of refrigerated storage, both traditional culture and Petrifilm® analysis demonstrated significantly higher bacterial growth in the control samples compared with curcumin-supplemented extenders. Samples supplemented with 0.25 mM (C2) and 0.5 mM (C3) curcumin showed the lowest bacterial counts in Petrifilm® plates. In traditional culture, samples supplemented with 0.125 mM curcumin (C1) also showed significantly lower bacterial growth compared with the control (Figure 6).

Overall, curcumin supplementation was associated with reduced cultivable aerobic bacterial growth during refrigerated storage compared with the control extender without curcumin, with more pronounced differences observed after prolonged storage.

## 4 Discussion

### 4.1 Effect of curcumin on sperm motility and viability: dose–response and toxicity

Refrigerated storage of equine spermatozoa is associated with a progressive decline in sperm quality, mainly due to cold shock, oxidative imbalance, and alterations of plasma and acrosomal membranes (4). In this study, curcumin was evaluated primarily to determine whether its antimicrobial properties could be exploited during storage without compromising basic sperm quality parameters. Under short-term refrigerated conditions, curcumin supplementation did not induce detectable changes in total motility, sperm viability, or acrosomal integrity, regardless of the concentration tested. This finding indicates that free curcumin does not exert acute toxic effects on equine epididymal spermatozoa during early cooling and that sperm function is initially preserved. The absence of early effects is consistent with the buffering capacity of equine semen extenders, which are designed to maintain sperm quality during the first phase of refrigerated storage (4, 5, 37).

In contrast, after prolonged storage, a clear dose and time dependent response became evident. Higher curcumin concentrations were associated with reduced sperm viability and acrosomal integrity, together with alterations in motility, whereas the lowest concentration tested did not differ from the control. These results suggest that curcumin may exert cumulative detrimental effects on spermatozoa when present at elevated concentrations for extended periods, rather than inducing immediate toxicity.

This concentration-dependent behavior is consistent with previous findings in the equine species. Rossi et al. (31) reported that low curcumin concentrations did not affect stallion sperm motility after short-term storage, whereas higher concentrations significantly reduced motility (44). Although storage conditions and experimental design differ, both studies support the existence of a threshold concentration above which curcumin becomes detrimental to equine spermatozoa, which may be related to a dose-dependent transition from antioxidant to pro-oxidant effects under specific storage conditions (45).

Curcumin is known to display a dual antioxidant and pro-oxidant behavior depending on concentration, exposure time, and cellular environment (30, 46). At higher concentrations, curcumin may interfere with membrane lipid organization, mitochondrial activity, or intracellular redox homeostasis, ultimately compromising sperm viability and membrane integrity. Importantly, none of the concentrations tested in the present study improved sperm quality parameters, indicating that, under refrigerated conditions, free curcumin should not be considered a sperm-protective additive but rather a compound with a limited safety window. Recent evidence further supports this interpretation. Nasiri-Foomani et al. (47) demonstrated that curcumin-loaded niosomal nanoparticles significantly improved motility, membrane functionality, and lipid peroxidation in cooled stallion semen, whereas free curcumin showed weaker or inconsistent effects.

These findings suggest that the antioxidant potential of curcumin may be constrained by its physico-chemical properties, such as limited aqueous solubility and stability, which could restrict its effective bioavailability under semen storage conditions. In this context, the lack of sperm-protective effects observed in the present study is consistent with the use of free curcumin at the concentrations tested.

### 4.2 Curcumin-associated changes in sperm motility patterns

Beyond total motility, computer-assisted sperm analysis revealed that curcumin supplementation influenced sperm movement characteristics in a concentration- and time-dependent manner (48, 49). Under short-term refrigeration, higher curcumin concentrations were associated with reduced velocity-related parameters and increased straightness, indicating slower but more rectilinear trajectories. While progressive movement is required for fertilization, reduced velocity may compromise sperm transport efficiency within the female reproductive tract (50).

After prolonged storage, these alterations became more pronounced. Exposure to the highest curcumin concentration resulted in marked reductions in velocity parameters together with decreased ALH and BCF, suggesting impaired flagellar beat amplitude and frequency. These changes are compatible with compromised axonemal function and altered energy metabolism, and they likely precede or accompany the observed decline in sperm viability and membrane integrity (51).

Together, these findings indicate that elevated curcumin concentrations do not simply reduce the proportion of motile spermatozoa but also alter the qualitative features of sperm movement, which may have functional implications for fertilization. This reinforces the importance of considering detailed kinematic parameters when evaluating the biological impact of extender additives.

### 4.3 Antimicrobial activity of curcumin during refrigerated storage

Semen is not a sterile biological fluid, and bacterial contamination is a well-recognized factor negatively affecting sperm viability, fertility potential, and storage lifespan (12, 37). During refrigerated storage, bacterial metabolism may contribute to the production of reactive oxygen species and other toxic by-products that impair sperm membrane integrity, mitochondrial activity, and motility (52). In addition, the use of contaminated semen has been associated with an increased risk of post-breeding endometritis in mares, highlighting the clinical importance of controlling microbial proliferation in semen preservation protocols (13).

In the present study, supplementation of an antibiotic-free extender with curcumin consistently reduced cultivable bacterial growth during refrigerated storage. This inhibitory effect was observed using both MacConkey agar and Petrifilm® Aerobic Count plates, indicating a genuine antimicrobial action of curcumin under the experimental conditions employed. The reduction in bacterial growth became more evident after prolonged refrigeration, supporting a cumulative antimicrobial effect over storage time.

Comparable antimicrobial effects of curcumin and other natural bioactive compounds have been previously reported in semen preservation systems across species (19, 34, 52, 53). Although curcumin is generally less potent than conventional antibiotics, its capacity to limit bacterial proliferation without contributing to antimicrobial resistance underscores its potential as a complementary strategy in semen extenders. Importantly, antimicrobial activity is likely less dependent on intracellular uptake and bioavailability than antioxidant protection, which may explain why free curcumin effectively reduced bacterial growth despite failing to improve sperm quality parameters in the present study (34).

The microbiological assessment in this study was focused on cultivable aerobic and facultative anaerobic bacteria, as bacterial growth was evaluated using MacConkey agar—primarily selective for Gram-negative microorganisms—and Petrifilm® Aerobic Count plates for total aerobic bacterial counts. This methodological approach is supported by previous studies reporting that Gram-negative aerobic and facultative anaerobic bacteria, particularly members of the Enterobacteriaceae family, constitute the predominant microbial population in equine semen during storage (12, 37, 52). Strict anaerobic bacteria were not evaluated under the culture and incubation conditions employed and are generally considered less prevalent in routinely collected and refrigerated semen samples. Consequently, the antimicrobial effects observed in the present study should be interpreted as referring mainly to the bacterial populations most associated {Citation} with semen contamination and quality deterioration during refrigerated storage.

### 4.4 Relevance of epididymal sperm as a model for microbiological and extender studies

Epididymal spermatozoa provide a valuable experimental model for microbiological studies, as they are antibiotic-naïve and typically exhibit lower and more homogeneous bacterial contamination than ejaculated semen (36, 41, 42). This allows the evaluation of antimicrobial additives without confounding effects from seminal plasma or prior antibiotic exposure.

Although epididymal sperm initially lack seminal plasma proteins involved in sperm activation, washing and dilution in appropriate extenders can partially restore motility and functionality (53, 54). Importantly, several studies have reported acceptable pregnancy rates using cooled or cryopreserved epididymal sperm in horses, supporting its relevance for assisted reproduction and genetic conservation (42).

Clinically, epididymal sperm recovery represents the only option for preserving genetic material in cases of sudden death or severe trauma. While ejaculated semen differs in composition and microbial complexity, the epididymal model provides a relevant and practical framework for evaluating antimicrobial strategies under controlled conditions.

## 5 Conclusion

The results of the present study indicate that curcumin supplementation in an antibiotic-free extender influences both bacterial growth and sperm quality in refrigerated equine epididymal sperm in a concentration- and time-dependent manner. Under the experimental conditions evaluated, curcumin did not improve sperm quality parameters at any of the concentrations tested.

Curcumin supplementation reduced cultivable aerobic bacterial growth during refrigerated storage, with more pronounced effects observed after prolonged storage. However, higher concentrations were associated with a deterioration of sperm viability, acrosomal integrity, and motility parameters after 96 h of refrigeration.

Among the concentrations evaluated, 0.125 mM curcumin provided the most favorable balance between antimicrobial activity and preservation of basic sperm quality parameters, without inducing detectable detrimental effects during storage. These findings suggest that low concentrations of curcumin may be suitable for further evaluation as a potential antimicrobial additive in semen extenders.

The use of epididymal spermatozoa highlights the clinical relevance of curcumin supplementation in scenarios where ejaculated semen cannot be obtained, such as post-mortem sperm recovery or conditions preventing ejaculation. Nevertheless, due to the lower initial bacterial load and the absence of seminal plasma in epididymal semen, extrapolation of these results to ejaculated semen used in routine artificial insemination should be performed with caution.

Taken together, the present results support the need for further studies to assess the suitability of curcumin as a complementary antimicrobial additive in equine semen preservation. Such studies should include ejaculated semen, comprehensive microbiological characterization, functional sperm assessments, and fertility trials.

## Data Availability

The original contributions presented in the study are included in the article/Supplementary material, further inquiries can be directed to the corresponding author.
