# Supplementary material for: Dose-dependent effects of curcumin on bacterial growth and sperm quality during refrigerated storage of equine epididymal sperm
Source: Front Vet Sci. 2026 Feb 9;13:1739360. doi: 10.3389/fvets.2026.1739360 (PMC12926836; doi:10.3389/fvets.2026.1739360)
Supplement: Supplementary file 1 [file Table_1.docx]

**Appendix Table**

**Table S1**: Statistical significance (p-value) of sperm motility, viability and acrosome integrity as influenced by study factors ( horse effect, curcumin addition, and the horse-curcumin interaction) at 1 and 96 Hours of Refrigeration.

| Tº | Factor | Total Motility (%) | Viability (%) | Acrosome Integrity (%) |
| --- | --- | --- | --- | --- |
| 1 h | **Horse** | <0.001 | <0.001 | <0.001 |
|  | **Curcumin** | 0.572 | 0.906 | 0.328 |
|  | **Horse * Curcumin** | 0.754 | 0.692 | 0.998 |
| 96 h | **Horse** | 0.237 | <0.001 | 0.022 |
|  | **Curcumin** | 0.007 | <0.001 | <0.001 |
|  | **Horse * Curcumin** | 0.008 | <0.001 | <0.001 |
